# Association between deep neural network-derived electrocardiographic-age and incident stroke

**DOI:** 10.3389/fcvm.2024.1368094

**Published:** 2024-06-28

**Authors:** Robert Leung, Biqi Wang, Matthew Gottbrecht, Adam Doerr, Neil Marya, Apurv Soni, David D. McManus, Honghuang Lin

**Affiliations:** ^1^ Program in Digital Medicine, Department of Medicine, UMass Chan Medical School, Worcester, MA, United States; ^2^ Division of Health Systems Science, Department of Medicine, UMass Chan Medical School, Worcester, MA, United States; ^3^ Division of Cardiology, Department of Medicine, UMass Chan Medical School, Worcester, MA, United States; ^4^ Division of Gastroenterology, Department of Medicine, UMass Chan Medical School, Worcester, MA, United States

**Keywords:** stroke, ECG-age, UK Biobank, AI, DNN

## Abstract

**Background:**

Stroke continues to be a leading cause of death and disability worldwide despite improvements in prevention and treatment. Traditional stroke risk calculators are biased and imprecise. Novel stroke predictors need to be identified. Recently, deep neural networks (DNNs) have been used to determine age from ECGs, otherwise known as the electrocardiographic-age (ECG-age), which predicts clinical outcomes. However, the relationship between ECG-age and stroke has not been well studied. We hypothesized that ECG-age is associated with incident stroke.

**Methods:**

In this study, UK Biobank participants with available ECGs (from 2014 or later). ECG-age was estimated using a deep neural network (DNN) applied to raw ECG waveforms. We calculated the Δage (ECG-age minus chronological age) and classified individuals as having normal, accelerated, or decelerated aging if Δage was within, higher, or lower than the mean absolute error of the model, respectively. Multivariable Cox proportional hazards regression models adjusted for age, sex, and clinical factors were used to assess the association between Δage and incident stroke.

**Results:**

The study population included 67,757 UK Biobank participants (mean age 65 ± 8 years; 48.3% male). Every 10-year increase in Δage was associated with a 22% increase in incident stroke [HR, 1.22 (95% CI, 1.00–1.49)] in the multivariable-adjusted model. Accelerated aging was associated with a 42% increase in incident stroke [HR, 1.42 (95% CI, 1.12–1.80)] compared to normal aging. In addition, Δage was associated with prevalent stroke [OR, 1.28 (95% CI, 1.11–1.49)].

**Conclusions:**

DNN-estimated ECG-age was associated with incident and prevalent stroke in the UK Biobank. Further investigation is required to determine if ECG-age can be used as a reliable biomarker of stroke risk.

## Introduction

Stroke is the second leading cause of death and disability worldwide despite improvements in prevention and treatment. It continues to pose a massive burden on both individual and societal levels. Stroke alone accounts for approximately 116 million global disability-adjusted life-years (DALYs) lost in 2016 and over US$891 billion in 2019 (1, 2). Globally, the prevalence of stroke is expected to rise, leading to further increases in stroke-related costs (3). In the US and other developed nations, the main driver for increased stroke prevalence is advanced age, as age is the most important non-modifiable risk factor. Moreover, the literature suggests that evidence-based guidelines for stroke care are less likely to be followed with older age (4). As a result, stroke risk reduction among older patients remains crucial.

Predicting which patients are at highest risk for a certain disease enable early intervention, thereby improving patient outcomes and reducing pressure on the healthcare system through primary prevention. However, at present many stroke risk calculators like the American College of Cardiology/American Heart Association (ACC/AHA) Pooled Cohort Equations (PCE) and the Framingham Stroke Risk Score (FSRS) are derived from conventional statistical methods that include a few predictors quantified by human observers. These models oversimplify complex relationships (5, 6). In contrast, a study by Dritsas and Trigka demonstrated the potential of various machine learning (ML) algorithms in predicting stroke (7), an approach that has been used to improve the prediction of other diseases like hypertension and diabetes mellitus (8, 9). Further, both the PCE and FSRS tend to underperform in certain ethnicities and socioeconomic classes. They also do not incorporate novel risk markers that some consider important for risk assessment (10). Fortunately, one of the key applications of machine learning (ML) in healthcare is risk stratification and clinical decision support. ML has the potential to circumvent these limitations and outperform current stroke risk prediction tools (5).

The ML method utilized depends on the type of data. Deep learning (DL) models based on neural networks have been used to extract features from imaging and ECGs as a basis for predicting cardiovascular disease (CVD) risk (5). One such model was a deep neural network (DNN)-based age prediction model developed on the Clinical Outcomes in Digital Electrocardiography (CODE) data set to predict an individual's age based on ECG waveform, otherwise known as the electrocardiographic age (ECG-age) (11). Existing literature has shown that DNN-estimated age is highly correlated to chronological age with the delta age (ECG-age minus chronological age) being a predictor of overall mortality (12). A recent study showed that advanced “biological aging,” as predicted by ECG data, was associated with increased risk of all-cause mortality, myocardial infarction (MI), atrial fibrillation (AF), and heart failure, highlighting the vital role of ECG as a biomarker of CVD risk and its potential role in stroke prediction (11, 13).

We hypothesize that ECG changes with age relate strongly to stroke risk. This study aimed to evaluate whether DNN-estimated ECG-age can predict incident stroke in the large-scale and long-term UK Biobank.

## Methods

### Study population

Our study population is comprised of more than half a million volunteers aged 40–69 years who enrolled in the UK Biobank between the years 2006 and 2010 (14). These participants lived within 25 miles of one of the 22 assessment centers located throughout England, Wales, and Scotland. On enrollment in the UK Biobank, participants provided signed consent and answered questions about sociodemographic, lifestyle, environmental, and health-related factors. 12-lead (at-rest) ECGs were performed at an imaging assessment center for UK Biobank. Exact details of the procedure can be found at https://biobank.ctsu.ox.ac.uk/crystal/refer.cgi?id=510. ECG acquisition was approximately 10 s per individual. Participants who had an ECG done during their clinic visit (2014 or later) or subsequent clinic visit (2019 or later) were included in our study. For participants with two ECGs, only the first valid ECG was accounted. We excluded individuals with prevalent stroke (*n* = 808), which left 67,757 individuals for the association analysis of ECG-age and incident stroke.

### Clinical variables

All the lifestyle and clinical risk factors of stroke were measured at the same time as the ECG measurement. Lifestyle factors (smoking, alcohol consumption, physical activity, and diet) were self-reported and were dichotomized as previous publications presented (15). For blood pressure, two measures were taken one minute apart using a digital blood pressure monitor. Body mass index was calculated as weight in kilograms divided by height in meters squared. Particulate matter (PM) air pollution (PM_2.5_, unit micro-g/m3) was collected from local environment air pollution data in the year 2010 (Data Field 24,006). Another air pollutant, PM_10_ (PM with an aerodynamic diameter of <10 micro-g), was measured in 2007 (Data Field 24,019) and 2010 (Data Field 24,005). Health-related outcomes including diabetes mellitus (DM), chronic kidney diseases (CKD), and dyslipidemia were followed up based on the primary care data (READ code), hospital inpatient data (ICD-9/ICD-10 codes), death registry (ICD-10 code), and self-reported medical conditions. Their first occurrence dates were derived and provided by the UK Biobank: DM included type 1, type 2, and other diabetes (Data Fields 130,706, 130,708, 130,710, 130,712, 130,714), CKD Data Field was 132,032, and dyslipidemia was 130,814.

### Study outcomes

Ischemic stroke and hemorrhagic (intracerebral and subarachnoid subtypes) stroke were the outcomes of interest. These outcomes were defined by self-reported medical conditions, ICD-9, or ICD-10 codes (16, 17). The Data Field related to the stroke outcomes were 42,006, 42,008, 42,010, and 42,012. The included participants were followed up since they took the first ECG until whichever came first: newly diagnosed stroke, or their last follow-up time in the UK Biobank, in which they were free of stroke, death, or end of August 2023.

### DNN-estimated ECG-age

The ECG-age was obtained using a DNN that applies the raw ECG waveform in an end-to-end approach as opposed to classical automatic analysis methods (18). The model was trained to predict a person's age by extracting features directly from the data instead of relying on traditional ECG interpretation (18, 19). Through this learning process, the DNN captured how aging affects ECG waveform (11). For this study, the raw ECG signals used to generate the ECG-age model were derived from the CODE study, which was further refined using the UK Biobank ECG data. The CODE study is part of the Telehealth Network of Minas Gerais (TNMG) with a database containing ECGs obtained from 2010 to 2017 in Brazilian primary care settings (20). To date, the CODE data set has been recognized as the world's largest ECG database used to develop artificial intelligence (AI)-ECG applications with 1,558, 415 participants (21). The ECG-age model in the CODE study and its development have been previously described (12). The model uses a DNN to make predictions like the residual network proposed for image classification except with unidimensional signals (11). More information on the code used in the DNN-estimated ECG-age model training, evaluation, and statistical analysis can be found at https://github.com/antonior92/ecg-age-prediction.

### Statistical analyses

To assess the DNN-estimated ECG-age as a predictor for stroke risk, we used the Δage (ECG-age minus the chronological age) as the independent variable and thereby capturing the excess risk caused by a greater decline in cardiovascular health than expected by chronological aging. We also separated participants into three aging categories based on Δage mean absolute error (MAE) (12). Those with an ECG-age older than the chronological age by ≥MAE years were considered accelerated aging. Those with an ECG-age younger than the chronological age by ≥MAE years were considered decelerated aging. Those with an ECG-age within the MAE were considered normal aging. We used Cox proportional hazards regression models to assess the association of Δage and incident stroke: the first model was chronological age and sex adjusted, and the second multivariable model was additionally adjusted for SBP, BMI, particulate matter air pollution, smoking status, diet, physical activity level, alcohol consumption, diabetes, CKD, and dyslipidemia. The results were expressed as stroke risk of Δage per 10 years.

For the secondary analysis, we assessed the association of Δage with prevalent stroke by logistic regression models and adjusted for chronological age, sex, and additional clinical risk factors. Several sensitivity analyses included: (A) we excluded participants with prevalent atrial fibrillation at the time of ECG measurement, (B) we assessed the 5-year stroke risk by the Cox model, (C) we assessed the ability of Δage to predict incident stroke using the C-statistics where we calculated the change in the predictive capacity with and without the Δage in the multivariable Cox models, and the net reclassification index was also generated to assess the prediction ability (22), (D) we explored the non-linear relationship of Δage and the hazard of incident stroke by using the fractional polynomials and splines.

In our sensitivity analysis, we performed stratified analysis in two age groups (chronological age <60, or ≥ 60), and in men and women separately to assess the associations of Δage and incident stroke. We used the multivariable-adjusted Kaplan–Meier curves which were achieved by the inverse probability weighting method to illustrate the cumulative risk of stroke across decelerated, normal, and accelerated aging groups. We used *P* values less than 0.05 (two-sided) as the statistical significance level. All the analyses were performed using R software package version 4.2.1 (https://www.r-project.org/).

## Results

Our primary analysis includes 67,757 participants (68,565 for the secondary analysis) from the UK Biobank. The mean age was 65 ± 8 years and 48.3% were men. The clinical characteristics are shown in Table 1. Those in the accelerated aging cohort were more likely male, had a higher BMI, and more current smoking, alcohol consumption, unhealthy diet, and less physical activity (Supplementary Table S1). ECG-age was generated with a DNN (based on a previous model from the CODE study) using raw ECG waveform data. On the other hand, participants within the decelerated age group had less prevalent DM, CKD, and dyslipidemia (Supplementary Table S1). A total of 808 participants had a stroke before or at the time an ECG was performed. During an average of 3.94 ± 2.55 years of follow-up, an additional 379 participants were diagnosed with stroke.

**Table 1:** Baseline characteristics of participants with ECG-age in the UK Biobank.

	Overall	No stroke	Incident stroke	*P* value
*n*	67,757	67,378	379	
Chronological age [mean (SD)]	65 (8)	65 (8)	68 (7)	<0.001
Men (%)	32,706 (48.3)	32,487 (48.2)	219 (57.8)	<0.001
Δage [mean (SD)]	−0.01 (5.88)	−0.02 (5.88)	1.09 (6.34)	<0.001
Body mass index [mean (SD)]	26.6 (4.5)	26.6 (4.5)	26.9 (4.4)	0.30
Systolic blood pressure [mean (SD)]	141 (19)	141 (19)	146 (21)	<0.001
Diastolic blood pressure [mean (SD)]	79 (10)	79 (10)	81 (12)	0.005
Current smoker (%)	2,273 (3.4)	2,259 (3.4)	14 (3.7)	0.82
Healthy drinking (%)	44,985 (66.9)	44,727 (66.9)	258 (68.8)	0.47
Physically active (%)	53,261 (87.2)	52,970 (87.2)	291 (86.4)	0.71
Healthy diet (%)	29,686 (45.0)	29,525 (45.0)	161 (43.9)	0.71
Prevalent diabetes (%)	3,846 (5.7)	3,807 (5.7)	39 (10.3)	<0.001
Prevalent chronic kidney diseases (%)	1,444 (2.1)	1,428 (2.1)	16 (4.2)	0.008
Prevalent dyslipidemia (%)	16,013 (23.6)	15,901 (23.6)	112 (29.6)	0.008
Incident ischemic stroke (%)	304 (0.4)	0 (0.0)	304 (80.2)	<0.001
Incident hemorrhagic stroke (%)	86 (0.1)	0 (0.0)	86 (22.7)	<0.001
Incident hemorrhagic intracerebral stroke (%)	65 (0.1)	0 (0.0)	65 (17.2)	<0.001
Incident hemorrhagic subarachnoid stroke (%)	32 (0.0)	0 (0.0)	32 (8.4)	<0.001
Follow-up years [mean (SD)]	3.94 (2.55)	3.95 (2.56)	2.75 (1.88)	<0.001

Δage, ECG predicted age—chronological age; *P* values were from the *t*-test (continuous variables) or Chi-square test (categorical variables).

### Association of Δage with incident stroke

Table 2 shows the association of Δage with incident stroke in the chronological age- and sex-adjusted, and multivariable-adjusted models. Every 10-year increase in Δage was associated with a 22% increase in the risk of incident stroke [HR = 1.22 (95% CI, 1.00–1.49)] after adjusting for multiple risk factors. The association remained significant if we restricted to 5 years risk of incident stroke in both age- and sex- adjusted and multivariable-adjusted models (Supplementary Table S2). The association of Δage with incident stroke in the multivariable-adjusted model was attenuated when we excluded prevalent atrial fibrillation cases (Supplementary Table S3). However, the inclusion of Δage did not significantly improve the prediction of incident stroke with a change of C-statistics of 0.003 [95% CI, −0.003–0.009] in the multivariable model. There was also no significant change in terms of net reclassification improvement (NRI) (*P* > 0.05).

**Table 2:** Associations of Δage (per 10 years) and incident stroke.

	Chronological age and sex adjusted model					Multivariable adjusted model				
	Total	Incident cases	HR (95% CI)	*P* value	*P* interact	Total	Incident cases	HR (95% CI)	*P* value	*P* interact
All	67,757	379	1.38 (1.17, 1.64)	<0.001	–	50,100	282	1.22 (1.00, 1.49)	0.05	–
Baseline chronological age <60	18,266	60	1.06 (0.68, 1.64)	0.81	0.18	14,009	46	0.82 (0.49, 1.38)	0.45	0.12
Baseline chronological age ≥60	49,491	319	1.45 (1.20, 1.74)	<0.001		36,091	236	1.31 (1.05, 1.63)	0.02	
Men	32,706	219	1.49 (1.19, 1.87)	<0.001	0.28	24,501	170	1.34 (1.03, 1.74)	0.03	0.25
Women	35,051	160	1.24 (0.96, 1.61)	0.11		25,599	112	1.07 (0.78, 1.47)	0.66	

Multivariable model adjusted for chronological age, sex, BMI, current smoking, drinking, physical activity, diet, systolic blood pressure, prevalent diabetes, prevalent CKD, and prevalent dyslipidemia.

**Figure 1:**
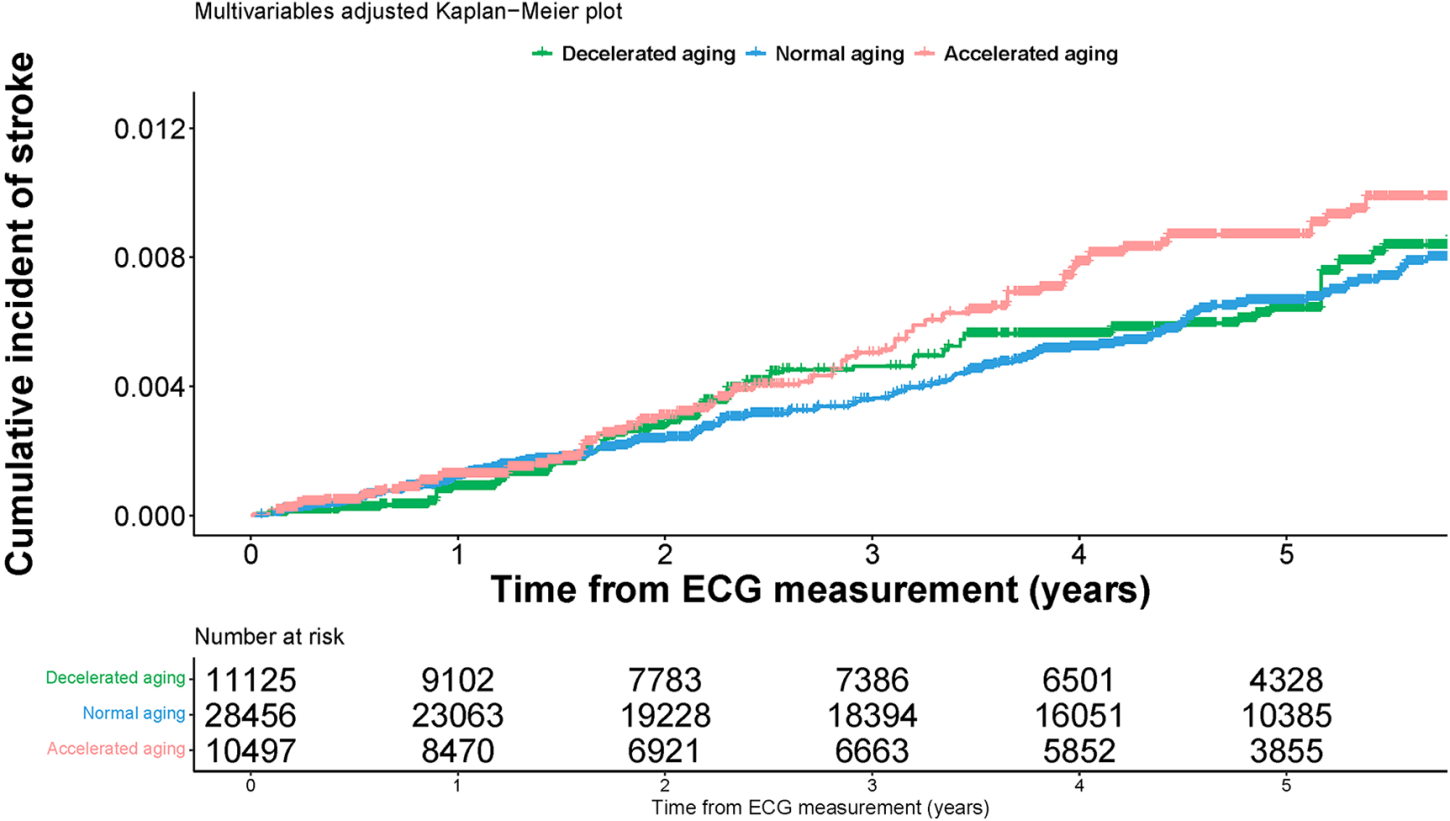
Multivariable adjusted Kaplan-Meier plot for the associations of ECG aging groups and cumulative risk of incident stroke.

Figure 1 shows the multivariable-adjusted cumulative risk of incident stroke across three aging groups. Participants in the accelerated aging group had a higher probability of developing stroke compared to the normal and decelerated aging groups (log-rank test *P* value = 0.02). We also fit a smoothing spline fit for the relative hazard of incident stroke as a function of Δage (Supplementary Figure S1). The graph did not support a nonlinear relationship between Δage and hazard of incident stroke (*P* = 0.07 for the nonlinear term).

Two additional analyses on comparisons of the stroke risk models based on PCE with or without ECG Δage and revised FSRS (R-FSRS) with or without ECG Δage were conducted. Although there was subtle improvement in the C statistics when Δage was added to both models, these findings were not statistically significant (Supplementary Table S8).

### Sensitivity analysis

In the age and sex-stratified analysis, Δage was positively associated with stroke incidence in individuals older than 60 years and in men (HR = 1.31 [95% CI, 1.05–1.63] and 1.34 [95% CI, 1.03–1.74] respectively). However, the difference did not reach the pre-defined significance threshold (both with interaction *P* values greater than 0.05). Similar association results were seen in the incidence of ischemic stroke (Supplementary Table S4). Because we had very limited incident hemorrhagic stroke cases, we did not find any association of Δage and hemorrhagic stroke (Supplementary Table S1). Moreover, in Supplementary Table S5, accelerated aging was associated with a 42% increase in incident stroke [HR = 1.42 (95% CI, 1.12–1.80)] in the age- and sex-adjusted model. Decelerated aging was associated with a 10% decrease in incident stroke, but this finding was not statistically significant.

### Association of Δage with prevalent stroke

As shown in Supplementary Table S6, participants with prevalent stroke were more likely to be male and have higher Δage, BMI, and systolic blood pressure. Like their incident stroke counterparts, they were more likely to have prevalent DM, CKD, and dyslipidemia. Supplementary Table S7 shows the association between Δage and prevalent stroke in the age- and sex-adjusted, and multivariable-adjusted models. There was a significant association between prevalent stroke and Δage [OR, 1.28 (95% CI, 1.11–1.49)], suggesting that those with a history of stroke were 28% more likely to exhibit accelerated aging.

## Discussion

In this study, we found that Δage (the difference between DNN-estimated ECG-age and chronological age) was associated with incident stroke in UK Biobank. ECG-age was also associated with prevalent stroke even after adjusting for known risk factors. These results indicate that ECG-age may reflect an accelerated compromise in cardiac electrical function (11). Accelerated aging was able to predict incident stroke in the age- and sex-adjusted model. All in all, our findings suggest that ECG-age is connected to stroke in the community.

DNNs and other AI-related technologies are being increasingly implemented in healthcare. These technologies not only have the potential to transform various aspects of patient care, but also some studies are already suggesting that AI can perform as well as if not better than humans at certain tasks (23). As mentioned previously, commonly used stroke risk stratification tools (i.e., PCE, FSRS) fail to incorporate newer risk markers including pro-BNP and left ventricular hypertrophy, which are associated with increased risk of stroke (24, 25). Moreover, traditional risk calculators like the PCE tend to under- or overestimate according to ethnicity, socioeconomic class, and presence of inflammatory states. Our results demonstrated that the addition of ECG Δage in risk models like the PCE and R-FSRS could improve their accuracy in stroke risk assessment.

Current literature is rife with examples demonstrating significant relationships between specific ECG features, age, and CVD risk. A study by van der Wall et al. found that the most important features for the prediction of physiologic age were T wave morphology indices in leads V4 and V5, and *P* wave amplitude in leads aVR and II (26). Recent findings in population-based studies suggest that abnormal *P* wave terminal force in lead I, a marker of left atrial abnormality, is strongly associated with incident stroke (27). Moreover, lower heart variability has been shown to be associated with increased risk of CV events and mortality (28).

Similarly, previous studies have linked AI-generated ECG-age to cardiovascular risk and outcomes. A study by Raghunath et al. incorporated a DNN-based model using ECG waveforms, which predicted all-cause mortality (29). The ECG-age model developed by the CODE study was associated with all-cause mortality in multiple validation cohorts (12). One of these is the ELSA-Brasil cohort study (Brazilian Longitudinal Study of Adult Health), wherein accelerated aging was able to predict 1-year overall mortality (12). Others have examined the connection between Δage and clinical outcomes. Chang et al. found that—compared to chronological age—older DNN-estimated ECG-age was correlated to all-cause mortality and CVD (30). Our study adds to these findings by demonstrating that ECG-age is also able to predict incident stroke in the community.

Unfortunately, since the DNN remains partly enigmatic in terms of interpretation, the ways in which ECG-age can explain cardiovascular risk may be complicated (11). Lima et al. found no significant differences between common ECG features (heart rate, P duration, QRS axis and duration, RR interval, and QTc interval) among subjects with accelerating, normal, or decelerating aging (12). To better understand this finding, the same study performed an analysis restricted to normal ECGs (per conventional standards) that reported a significant relationship between ECG-age and death. It appears that ECG-age prediction does not solely rely on traditional ECG abnormalities. This hypothesis is further corroborated by studies merging traditional and deep learning methods, suggesting that traditional ECG features alone do not fully explain the age prediction (19). Interestingly, DNN-estimated ECG-age appears to be a proxy for biological aging stemming from a single input, possibly capturing the residual risk from traditional and unknown risk factors (11). However, this lack of explainability could potentially be mitigated by incorporating aspects of the Bayesian approach. This approach has been used to infer ‘heart age’ from a patient's chronological age and sex by assessing and quantifying an individual's expected vs. actual ECG findings (31). Various ECG parameters were used as modifiers of the Bayesian-predicted heart age including QRS-T angle, a strong prognostic factor for all-cause mortality and CV events (31, 32).

There are a few limitations to address in this study. First, use of deep learning does not allow for feature extraction or explainability, leading to uncertainty about how ECG-age detects stroke risk. Future research should concentrate on understanding the components associated with DNN-estimated ECG-age and investigating if ECG-age quantifies modifiable excess risk amenable to early intervention (11). Moreover, our study population was predominantly healthy, better educated, and of white European descent. On the other hand, the DNN model used to predict ECG-age in our study was trained by extracting features from the CODE dataset, which is derived from a population that is generally sicker than the UK Biobank cohort. Also, compared to the hold-out split or CODE-15% cohort, mean age and the prevalence of CV risk factors were higher in both validation cohorts used in the CODE study. As such, the generalizability of our results to more diverse populations is unknown. Lastly, we cannot infer the causality of ECG-age to stroke due to the observational nature of our study and potential residual confounding factors.

In conclusion, DNN-estimated ECG-age was associated with incident and prevalent stroke in a large population-based study. Further analysis is needed to determine if ECG-age can be used as a practical biomarker of stroke risk.

## Data Availability

The original contributions presented in the study are included in the article/**Supplementary Material**, further inquiries can be directed to the corresponding author.
