# Supplementary material for: Association between deep neural network-derived electrocardiographic-age and incident stroke
Source: Front Cardiovasc Med. 2024 Jun 28;11:1368094. doi: 10.3389/fcvm.2024.1368094 (PMC11239432; doi:10.3389/fcvm.2024.1368094)
Supplement: Supplementary file 1 [file Datasheet1.docx]

**Supplement**

**Table S-1.** Baseline characteristics of participants with ECG-age by categorical ECG aging groups.

|  | Decelerated aging | Normal aging | Accelerated aging | P value |
| --- | --- | --- | --- | --- |
| n | 14,644 | 38,642 | 14,471 |  |
| Chronological age (mean (SD)) | 65 (8) | 65 (8) | 65 (8) | <0.001 |
| Men (%) | 5683 (38.8) | 18449 (47.7) | 8574 (59.2) | <0.001 |
| Δage (mean (SD)) | -7.87 (2.55) | -0.09 (2.63) | 8.14 (2.82) | <0.001 |
| Body mass index (mean (SD)) | 25.7 (4.2) | 26.7 (4.5) | 27.4 (4.8) | <0.001 |
| Systolic blood pressure (mean (SD)) | 137 (19) | 141 (19) | 145 (19) | <0.001 |
| Diastolic blood pressure (mean (SD)) | 77 (10) | 79 (10) | 82 (10) | <0.001 |
| Current smoker (%) | 363 (2.5) | 1270 (3.3) | 640 (4.5) | <0.001 |
| Healthy drinking (%) | 9939 (68.4) | 25736 (67.1) | 9310 (64.9) | <0.001 |
| Physically active (%) | 11861 (88.4) | 30255 (87.0) | 11145 (86.4) | <0.001 |
| Healthy diet (%) | 6905 (48.3) | 16804 (44.6) | 5977 (42.6) | <0.001 |
| Prevalent diabetes (%) | 630 (4.3) | 2115 (5.5) | 1101 (7.6) | <0.001 |
| Prevalent chronic kidney diseases (%) | 286 (2.0) | 810 (2.1) | 348 (2.4) | 0.02 |
| Prevalent dyslipidemia (%) | 3116 (21.3) | 9022 (23.3) | 3875 (26.8) | <0.001 |
| Incident stroke (%) | 71 (0.5) | 200 (0.5) | 108 (0.7) | 0.003 |
| Incident ischemic stroke (%) | 54 (0.4) | 160 (0.4) | 90 (0.6) | 0.002 |
| Incident hemorrhagic stroke (%) | 21 (0.2) | 46 (0.1) | 19 (0.2) | 0.77 |
| Incident hemorrhagic intracerebral stroke (%) | 15 (0.1) | 35 (0.1) | 15 (0.1) | 0.87 |
| Incident hemorrhagic subarachnoid stroke (%) | 8 (0.1) | 16 (0.0) | 8 (0.1) | 0.72 |
| Follow-up years (mean (SD)) | 4.09 (2.59) | 3.93 (2.53) | 3.83 (2.56) | <0.001 |

P values were from the t-test (continuous variables) or Chi-squared test (categorical variables).

Decelerated, normal, and accelerated aging was defined by the reported age ± MAE.

**Table S-2.** Associations of Δage (per 10 years) and risk of 5-year incident stroke by age and sex.

|  | Chronological age and sex adjusted model | | | | | Multivariable adjusted model | | | | |
| --- | --- | --- | --- | --- | --- | --- | --- | --- | --- | --- |
|  | Total | Incident cases in 5 years | HR (95% CI) | P value | P interact | Total | Incident cases in 5 years | HR (95% CI) | P value | P interact |
| **Stroke** | | | | | | | | | | |
| All | 67757 | 325 | 1.43 (1.19, 1.72) | <0.001 | - | 50100 | 235 | 1.25 (1.00, 1.56) | 0.05 | - |
| Baseline chronological age < 60 | 18266 | 45 | 1.24 (0.74, 2.06) | 0.41 | 0.54 | 14009 | 34 | 0.97 (0.53, 1.77) | 0.91 | 0.32 |
| Baseline chronological age ≥ 60 | 49491 | 280 | 1.46 (1.20, 1.78) | <0.001 |  | 36091 | 201 | 1.30 (1.03, 1.65) | 0.03 |  |
| Men | 32706 | 189 | 1.50 (1.18, 1.92) | 0.001 | 0.51 | 24501 | 144 | 1.34 (1.01, 1.78) | 0.04 | 0.39 |
| Women | 35051 | 136 | 1.33 (1.00, 1.77) | 0.05 |  | 25599 | 91 | 1.13 (0.79, 1.60) | 0.51 |  |
| **Ischemic stroke** | | | | | | | | | | |
| All | 68158 | 268 | 1.47 (1.20, 1.80) | <0.001 | - | 50388 | 191 | 1.25 (0.98, 1.60) | 0.07 | - |
| Baseline chronological age < 60 | 18325 | 36 | 1.06 (0.60, 1.88) | 0.84 | 0.23 | 14058 | 27 | 0.83 (0.42, 1.63) | 0.59 | 0.15 |
| Baseline chronological age ≥ 60 | 49833 | 232 | 1.54 (1.24, 1.92) | <0.001 |  | 36330 | 164 | 1.33 (1.02, 1.73) | 0.03 |  |
| Men | 32946 | 163 | 1.58 (1.21, 2.04) | <0.001 | 0.39 | 24676 | 124 | 1.44 (1.06, 1.95) | 0.02 | 0.12 |
| Women | 35212 | 105 | 1.32 (0.95, 1.82) | 0.10 |  | 25712 | 67 | 0.97 (0.65, 1.47) | 0.90 |  |

**Table S-3.** Associations of Δage (per 10 years) and incident stroke by age and sex after excluding prevalent atrial fibrillation.

|  | **Chronological age and sex adjusted model** | | | | | **Multivariable adjusted model** | | | | |
| --- | --- | --- | --- | --- | --- | --- | --- | --- | --- | --- |
|  | **Total** | **Incident cases** | **HR (95% CI)** | **P value** | **P interact** | **Total** | **Incident cases** | **HR (95% CI)** | **P value** | **P interact** |
| **Stroke** | | | | | | | | | | |
| All | 65471 | 349 | 1.26 (1.05, 1.51) | 0.01 | - | 48435 | 263 | 1.10 (0.89, 1.36) | 0.36 | - |
| Baseline chronological age < 60 | 18042 | 60 | 1.06 (0.68, 1.66) | 0.78 | 0.39 | 13837 | 46 | 0.83 (0.49, 1.39) | 0.47 | 0.24 |
| Baseline chronological age ≥ 60 | 47429 | 289 | 1.30 (1.07, 1.58) | 0.008 |  | 34598 | 217 | 1.16 (0.92, 1.46) | 0.20 |  |
| Men | 31019 | 198 | 1.31 (1.03, 1.67) | 0.03 | 0.57 | 23317 | 156 | 1.16 (0.88, 1.52) | 0.21 | 0.53 |
| Women | 34380 | 151 | 1.19 (0.91, 1.56) | 0.21 |  | 25118 | 107 | 1.03 (0.75, 1.43) | 0.66 |  |
| **Ischemic stroke** | | | | | | | | | | |
| All | 65841 | 281 | 1.30 (1.06, 1.58) | 0.01 | - | 48703 | 209 | 1.11 (0.88, 1.41) | 0.38 | - |
| Baseline chronological age < 60 | 18099 | 45 | 1.00 (0.60, 1.67) | 0.99 | 0.29 | 13885 | 34 | 0.77 (0.42, 1.41) | 0.40 | 0.20 |
| Baseline chronological age ≥ 60 | 47742 | 236 | 1.36 (1.09, 1.69) | 0.006 |  | 34818 | 175 | 1.18 (0.92, 1.53) | 0.20 |  |
| Men | 31312 | 167 | 1.34 (1.04, 1.74) | 0.03 | 0.64 | 23481 | 131 | 1.20 (0.89, 1.62) | 0.22 | 0.36 |
| Women | 34529 | 114 | 1.23 (0.90, 1.67) | 0.20 |  | 25222 | 78 | 0.98 (0.67, 1.43) | 0.90 |  |

**Table S-4.** Association of ECG-age and incident ischemic stroke in the UK Biobank.

|  | **Chronological age and sex adjusted model** | | | | | **Multivariable adjusted model** | | | | |
| --- | --- | --- | --- | --- | --- | --- | --- | --- | --- | --- |
|  | **Total** | **Incident cases** | **HR (95% CI)** | **P value** | **P interact** | **Total** | **Incident cases** | **HR (95% CI)** | **P value** | **P interact** |
| All | 68158 | 308 | 1.43 (1.18, 1.72) | <0.001 | - | 50388 | 226 | 1.24 (0.99, 1.55) | 0.06 | - |
| Baseline chronological age < 60 | 18325 | 45 | 0.99 (0.59, 1.65) | 0.97 | 0.14 | 18325 | 45 | 0.76 (0.42, 1.39) | 0.38 | 0.11 |
| Baseline chronological age ≥ 60 | 49833 | 263 | 1.51 (1.23, 1.86) | <0.001 |  | 36330 | 192 | 1.33 (1.05, 1.70) | 0.02 |  |
| Men | 32946 | 186 | 1.56 (1.22, 1.99) | <0.001 | 0.26 | 24676 | 144 | 1.43 (1.08, 1.89) | 0.01 | 0.10 |
| Women | 35212 | 122 | 1.25 (0.93, 1.69) | 0.15 |  | 25712 | 82 | 0.97 (0.67, 1.41) | 0.89 |  |

Multivariable model adjusted for chronological age, sex, BMI, current smoking, physical activity, drinking, diet, systolic blood pressure, prevalent diabetes, prevalent CKD, and prevalent dyslipidemia.

**Table S-5.** Associations of ECG aging groups and incident stroke.

|  | Chronological age and sex adjusted model | | Multivariable adjusted model | |
| --- | --- | --- | --- | --- |
|  | HR (95% CI) | P value | HR (95% CI) | P value |
| Stroke | | | | |
| Normal aging | Ref | Ref | Ref | Ref |
| Decelerated aging | 0.90 (0.67, 1.18) | 0.45 | 0.90 (0.66, 1.23) | 0.50 |
| Accelerated aging | 1.42 (1.12, 1.80) | 0.003 | 1.18 (0.85, 1.56) | 0.25 |
| Ischemic stroke | | | | |
| Normal aging | Ref | Ref | Ref | Ref |
| Decelerated aging | 0.89 (0.66, 1.21) | 0.47 | 0.89 (0.62, 1.26) | 0.50 |
| Accelerated aging | 1.47 (1.14, 1.90) | 0.003 | 1.16 (0.85, 1.58) | 0.35 |

**Table S-6**. Characteristics of participants of ECG-age and prevalent stroke.

|  | Without stroke | Prevalent stroke | P value |
| --- | --- | --- | --- |
| n | 67,757 | 808 |  |
| Chronological age (mean (SD)) | 65 (8) | 69 (7) | <0.001 |
| Men (%) | 32706 (48.3) | 531 (65.7) | <0.001 |
| Δage (mean (SD)) | -0.01 (5.88) | 1.15 (6.16) | <0.001 |
| Body mass index (mean (SD)) | 26.6 (4.5) | 27.4 (4.4) | <0.001 |
| Systolic blood pressure (mean (SD)) | 141 (19) | 143 (19) | 0.008 |
| Diastolic blood pressure (mean (SD)) | 79 (10) | 78 (10) | 0.07 |
| Current smoker (%) | 2273 (3.4) | 33 (4.2) | 0.26 |
| Healthy drinking (%) | 44985 (66.9) | 538 (67.5) | 0.76 |
| Physically active (%) | 53261 (87.2) | 589 (85.7) | 0.29 |
| Healthy diet (%) | 29686 (45.0) | 319 (40.9) | 0.03 |
| Prevalent diabetes (%) | 3846 (5.7) | 105 (13.0) | <0.001 |
| Prevalent chronic kidney diseases (%) | 1444 (2.1) | 44 (5.5) | <0.001 |
| Prevalent dyslipidemia (%) | 16013 (23.6) | 462 (57.2) | <0.001 |

**Table S-7.** Associations of Δage and prevalent stroke.

| Outcome | Chronological age and sex adjusted model | | Multivariable adjusted model | |
| --- | --- | --- | --- | --- |
|  | OR (95% CI) | P value | OR (95% CI) | P value |
| Prevalent stroke | 1.30 (1.15, 1.46) | <0.001 | 1.28 (1.11, 1.49) | <0.001 |

**Table S-8.** Stroke risk predictions with or without ECG Δage.

| Stroke risk model | C statistics (95% CI) | Stroke risk model | C statistics (95% CI) |
| --- | --- | --- | --- |
| Model A: PCE score only | 0.659 (0.626, 0.692) | Model A: R-FSRS score only | 0.685 (0.655, 0.715) |
| Model B: PCE + Δage | 0.668 (0.635, 0.701) | Model B: R-FSRS + Δage | 0.688 (0.658, 0.718) |
| C stats differences  (Model A - Model B) | 0.009 (-0.004, 0.021) | C stats differences  (Model A - Model B) | 0.003 (-0.003, 0.01) |


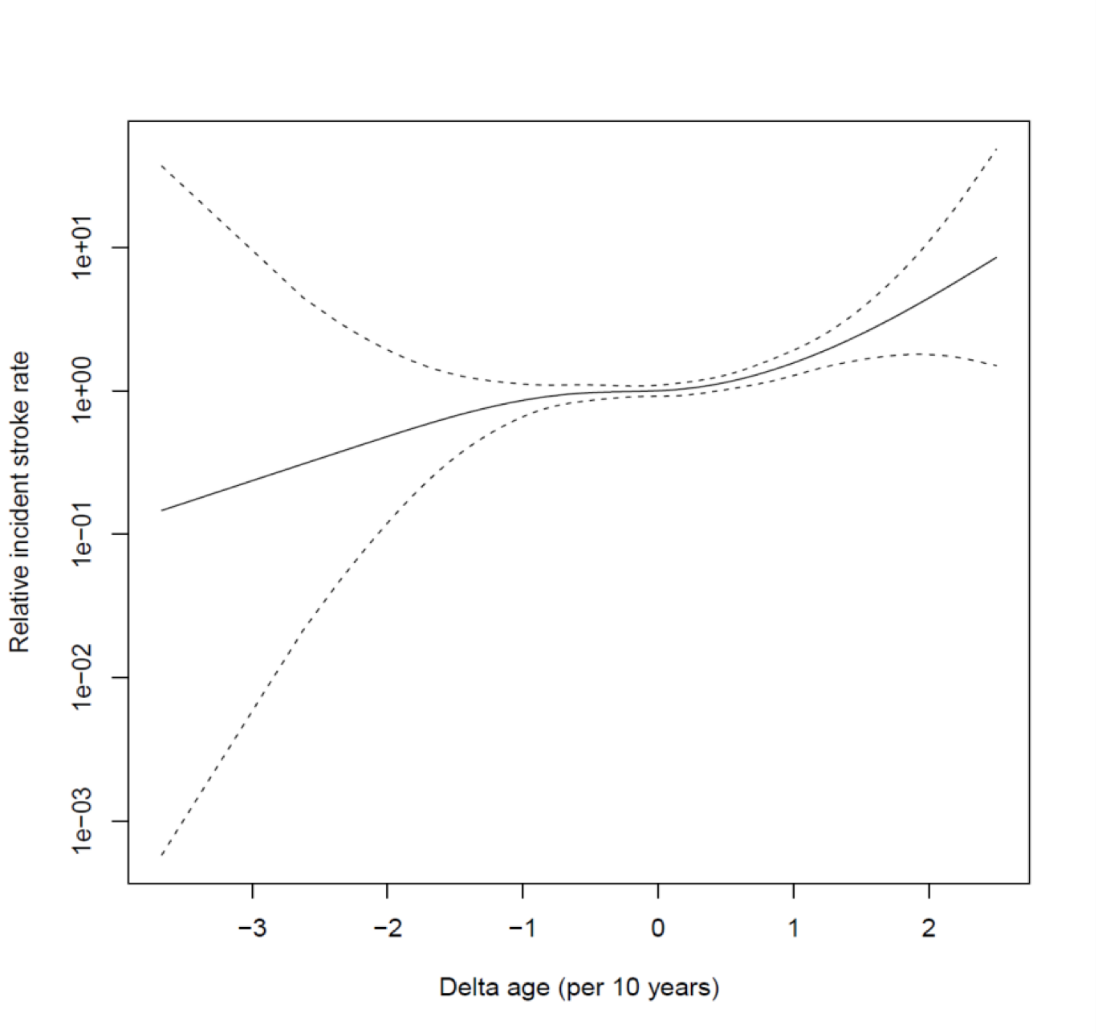


The delta age = 0.00013 as reference; P value = 0.069 for a nonlinear term.

**Figure S-1.** Smooth spline for Δ age and relative incident stroke rate.
